# Smart Soft Sensor Design with Hierarchical Sampling Strategy of Ensemble Gaussian Process Regression for Fermentation Processes

**DOI:** 10.3390/s20071957

**Published:** 2020-03-31

**Authors:** Xiaochen Sheng, Junxia Ma, Weili Xiong

**Affiliations:** 1 Key Laboratory of Advanced Process Control for Light Industry of Ministry of Education, Jiangnan University, Wuxi 214122, China; 6181905039@stu.jiangnan.edu.cn (X.S.); jxma@jiangnan.edu.cn (J.M.); 2 School of Internet of Things Engineering, Jiangnan University, Wuxi 214122, China

**Keywords:** Industrial 4.0, soft sensor, ensemble learning, active learning, hierarchical sampling, fermentation processes

## Abstract

Accurate and real-time quality prediction to realize the optimal process control at a competitive price is an important issue in Industrial 4.0. This paper shows a successful engineering application of how smart soft sensors can be combined with machine learning technique to significantly save human resources and improve performance under complex industrial conditions. Ensemble learning based soft sensors succeed in capturing complex nonlinearities, frequent dynamic changes, as well as time-varying characteristics in industrial processes. However, local model regions under traditional ensemble modelling methods are highly dependent on labeled data samples and, hence, their prediction accuracy might get affected when labeled samples are limited. A novel active learning (AL) framework upon the ensemble Gaussian process regression (GPR) model is proposed for smart soft sensor design in order to overcome this drawback. Firstly, to iteratively select the most informative unlabeled samples for labeling with hierarchical sampling based AL strategy, to then apply Gaussian mixture model (GMM) technique to autonomously identify operation phases, to further construct local GPR models without human involvement, and finally to integrate the base predictors by applying the Bayesian fusion strategy. Comparative studies for the penicillin fermentation process demonstrate the reliability and superiority of the recommended smart soft sensing. The cost of human annotation can be dramatically reduced by at least half while the prediction performance simultaneously keeps high.

## 1. Introduction

In recent years, artificial intelligence (AI) and machine learning (ML) have contributed to the great advancement of the Industry 4.0 [1,2]. It aims to ensure the high-quality control of production-based industries in the increasingly complex environment, such as the increased process automation, more efficient data analysis, lower human effort, safer working environment, and so on. Trillions of objects are connected to the Internet of Things (IoT), emerging huge amounts of data. Sensors play an important role in real-time and efficient data collection and processing. In industrial plants, many key variables are closely related to the product or process qualities but they can hardly be measured online with conventional hardware sensors. Soft sensors, which aim to realize the real-time prediction of these desired variables at low costs via constructing a satisfactory inference model between quality variables (usually are difficult-to-measure) and input variables (usually are easily accessible with sensors), have attracted growing attention in many industrial applications [3,4]. The data-driven algorithms based soft sensors are much more advantageous and easier for construction with litter process knowledge, which bring great convenience to the autonomous and intelligent control in industrial plants when compared with the first-principle model-based soft sensors that heavily rely on the prior knowledge and human experiences [4,5,6,7]. At present, plenty of multivariate statistical regression techniques such as partial least squares (PLS) [7,8], principal component regression (PCR) [8] models, and ML based techniques such as artificial neural network (ANN) [8], support vector machine (SVM) [9,10], and Gaussian process regression (GPR) [11,12,13,14] have been introduced to soft sensing.

However, many traditional nonlinear soft sensors tend to construct a desired global model for the estimation, which may perform poorly for processes with strong nonlinear and highly varying characteristics in the wide operation ranges. Ensemble learning methodology is proposed in order to effectively improve the generalization ability of the single predictor, and it has revealed great superiority in stability improvement [14,15,16]. The first step for ensemble model design is to construct a set of individual ensemble components. Several popular component generation approaches are bagging, boosting, clustering, and the subspace method [17,18]. Many clustering-based methods, which aim to divide the dataset into different clusters by exploring the internal structure of the objects and the relationship between them, have been verified to be practically and theoretically useful, such as K-means, expectation maximization (EM), fuzzy C-means (FCM), and Gaussian mixture model (GMM) [19,20,21,22]. Generally, given enough weighted Gaussian-distributed mixture components, the GMM technique makes it successful to smoothly approximate any given non-Gaussian probability density, and each component is considered as a suitable mode that can effectively represent the local distribution [22]. The prediction combination mechanism is the other step of the ensemble learning, whose criterions include simple averaging rule, weighted averaging combination, stacking strategy, Bayesian posterior probability, and so on [12,13,22,23]. The Bayesian fusion strategy has been proved to be naturally fit for model combination because of its strong statistical learning ability and efficient utilization for the collected dataset [13]. It contributes to better stability achievement by reducing the estimation variance.

However, a practical difficulty that is encountered in traditional ensemble modelling methods is the effective utilization of unlabeled dataset. When compared with process variables, the acquirement of key quality variables is much costlier and more time-consuming, as it always needs significant human expect, expensive measure instruments, or laboratory analyses [7,24]. Therefore, the historic dataset that was collected from industrial processes would contain a large number of unlabeled samples, which just consist of process variables. This unlabeled data that contain rich process information, if utilized effectively, might greatly advance the development of soft sensing and intelligent process control in Industry 4.0. Traditional semi-supervised learning techniques, including self-training methods, co-training methods, probabilistic generative modelling methods, and graph-based methods [15], could greatly enhance the generalization behavior of models by exploiting the unlabeled samples, but it also leads to some issues, such as the increase of computational effort and model instability. Besides, this method directly utilizes unlabeled samples to facilitate the learning process without any knowledge of human experts, while the designed model structure greatly influenced the improvement degree [12]. Hence, we intend to construct a smart modelling framework for ensemble learning method, under which both data information and process engineer knowledge can be driven for the soft sensor. 

Fortunately, the active learning (AL) technique shows great effectiveness and superiority in making full use of process dataset, by iteratively selecting valuable unlabeled samples for labeling with the knowledge of human experts. Therefore, the estimation capabilities of the AL based soft sensors can be effectively improved with the minimum time cost and human resource [24,25,26,27]. For the AL process, the most crucial issue is to determine a criterion that can effectively evaluate the potential quality of each unlabeled data point. Generally, the most meaningful unlabeled data, which consist of useful process information, are expected to be selected for labeling. However, many existing soft sensors under AL framework only consider the information of unlabeled samples and ignore the distribution information and spatial connectivity among them, which might result in more than one sample being selected in one small area during each learning iteration. In fact, it is unnecessary to select all of them, as they are likely to share same process information. The hierarchical clustering (HC) method provides a feasible approach for exploring spatial information between samples and their neighborhoods [28,29]. Compared with partition clustering algorithms, one of the most important advantages of this algorithm is that it can clearly show the clustering of dataset at different spatial levels [30]. The spatial information can be effectively extracted and utilized by pruning the HC tree with the AL strategy in order to mitigate the data sample selection problem.

Therefore, the motivation of this paper is to design a superior smart soft sensor, which can be referred as ensemble GPR model with hierarchical sampling based AL strategy (AL-EGPR), expecting to positively support the real-time data processing and process control in 4.0 industrial environments. The limitation of traditional supervised learning based regression methods raises nontrivial concerns regarding the efficient utilization of large amounts of unlabeled data. Subsequently, a novel AL strategy is proposed and incorporated into soft sensor modelling method. With the hierarchical sampling strategy, if the new unlabeled sample does not fall into any existing high-density clusters, it is considered to be highly informative and representative. In such cases, a desired number of most dissimilar unlabeled samples can be selected and used for manual annotation in each learning iteration, and, after that, added into the training dataset for the next model construction, until achieving the satisfactory accuracy, or all unlabeled samples have been labeled. Ensemble learning based on GPR model is further introduced to robust soft sensor design, aiming to achieve better generalization than single model-based predictors. Here, we choose the GPR model as the ensemble member as its characteristic probabilistic structure as well as the strong ability to handle abrupt changes and nonlinearity of industrial processes. In this method, the newly updated labeled training dataset is firstly divided into several different local data domains that can be realized by applying the GMM method, and multiple local GPR sub-models can be built for these sub-datasets, respectively. Afterwards, we introduce the Bayesian inference strategy to estimate the posterior probability of each query data sample with respect to local sub-models. Afterwards, the local predictions of GPR sub-models are effectively integrated into final prediction results by applying the finite mixture mechanism. Besides, the Bayesian information criterion (BIC) [18,20] is applied to determine the optimal number of GMM components in attempting to reduce the soft model complexity and enhance estimation ability. The recommended soft sensor has been applied to the prediction of penicillin concentration in the penicillin fermentation process, demonstrating that the high performance can be achieved at a low cost, in terms of the estimation accuracy and converge speed.

The remaining parts of this paper are structured, as follows. Section 2 briefly revisits the principle of the GPR model and GMM method. Section 3 presents the detailed methodology of the AL strategy, including the HC method and adaptive sampling strategy. Section 4 develops the ensemble GPR model based soft sensing technique with the AL strategy. Section 5 evaluates the effectiveness of the AL-EGPR method via the simulation results in an industrial process, and Section 6 concludes this paper.

## 2. Preliminaries

### 2.1. Gaussian Process Regression

A collection of random variables that all have a joint unknown Gaussian distribution can be significantly seen as a Gaussian process (GP), which has been greatly applied in order to define the desired distribution of flexible models in the field of regression and classification [12,13]. Given the training dataset of *m*-dimensional variable $X\left(n\times m\right)={\left[x_{1},x_{2},\cdots ,x_{n}\right]}^{T}$ and $y\left(n\times 1\right)={\left[y_{1},y_{2},\cdots ,y_{n}\right]}^{T}$, the output observations with zero-mean Gaussian prior distribution can be represented by:

(1) $$y=\left[f\left(x_{1}\right),f\left(x_{2}\right),\cdots ,f\left(x_{n}\right)\right]∼GP\left(0,K\right),$$

where GP(0,K) denotes the GP with zero-mean and **K**-covariance characteristics, while the *ij*-th element in matrix **K** is correspondingly described by kernel function $k\left(x_{i},x_{j}\right)$. In this research, squared-exponential function is used as the desired kernel function, which is defined as:

(2) $$k\left(x_{i},x_{j}\right)=\sigma_{f}^{2}\exp\left[-\frac{1}{2}{\left(x_{i}-x_{j}\right)}^{T}M\left(x_{i}-x_{j}\right)\right]+\delta_{i\;j}\sigma_{n}^{2},$$

with the unknown positive hyperparameter set $\Theta =\{l,\sigma_{f}^{2},\sigma_{n}^{2}\}$, where l denotes length-scale, $M=l^{-2}I$, $\sigma_{f}^{2}$, and $\sigma_{n}^{2}$ represent the signal variance and noise variance, respectively, while $\delta_{i\;j}$ is the Kronecker operator satisfying $\delta_{i\;j}=1$ if given i=j, otherwise, $\delta_{ij}=0$.

Therefore, the aim of GPR training process is to estimate the hyperparameter set Θ. A log-likelihood function maximization process can be performed to realize the parameter determination, which is represented, as follows:

(3) $$\begin{array}{ll} L\left(\Theta \right) & =\log p\left(y|X\right)\\ & =-\frac{n}{2}\log\left(2\pi \right)-\frac{1}{2}\log\left(\det\left(K\right)\right)-\frac{1}{2}y^{T}K^{-1}y \end{array}$$

(4) $$\begin{array}{ll} \Theta^{*} & =\left[l^{*},\sigma_{f}^{2*},\sigma_{n}^{2*}\right]=\underset{\Theta }{\arg\;\max}\log p\left(y|X,\Theta \right)\\ & =\underset{\Theta }{\arg\;\max}\log\int p\left(y|f,\sigma_{n}^{2}\right)p\left(X|\sigma_{f}^{2},l\right) \end{array}$$

Once the optimal hyperparameter set $\Theta^{*}$ is estimated, the GPR model can give an accurate estimation result regarding the distribution of quality variable ${\hat{y}}_{t}$ for the new test sample $x_{t}$, which is formulated as:

(5) $$p\left({\hat{y}}_{t}|x_{t}\right)=\int p\left[{\hat{y}}_{t}|f\left(x_{t}\right)\right]p\left[f\left(x_{t}\right)|x_{t}\right]df\left(x_{t}\right).$$

The posterior distribution of GPR output can be expressed by $\left({\hat{y}}_{t}|X,y,x_{t})∼N(\mu ({\hat{y}}_{t}),\sigma {\left({\hat{y}}_{t}\right)}^{2}\right)$, where $\mu ({\hat{y}}_{t})$ and $\sigma {\left({\hat{y}}_{t}\right)}^{2}$ denote the posterior mean and the variance of multivariate Gaussian distribution, respectively. In this case, we can describe the estimation results by:

(6) $$\mu ({\hat{y}}_{t})=k_{t}^{T}K^{-1}y,$$

(7) $$\sigma {\left({\hat{y}}_{t}\right)}^{2}=k(x_{t},x_{t})-k_{t}^{T}K^{-1}k_{t},$$

where $k_{t}={\left[k\left(x_{t},x_{1}\right),k\left(x_{t},x_{2}\right),\cdots ,k\left(x_{t},x_{n}\right)\right]}^{T}$ is the covariance vector matrix between data point $x_{t}$ and training points $x_{1:n}$. Finally, the expectation $\mu ({\hat{y}}_{t})$ of the present posterior distribution can be regarded as the estimation result ${\hat{y}}_{t}$ of the GPR based predictor.

### 2.2. Gaussian Mixture Model

GMM is commonly employed as an effective probabilistic modelling tool for the sake of approximating the data distribution, which is under the assumption that the distributions of all the data samples can be well approximated by the multivariate Gaussian mixture [21]. Given dataset X(n×m), which is assumed to follow a *K*-component Gaussian mixture distribution, we suppose that its probability density function is written as:

(8) $$p(X|\Xi )=\sum_{k=1}^{K}\pi_{k}p(X|\theta_{k})=\prod_{i=1}^{n}\sum_{k=1}^{K}\pi_{k}p(x_{i}|\theta_{k}),$$

where *K* is the number of Gaussian components, $\pi_{k}$ denotes the prior probability of the *k*th component and it subjects to $\sum_{k=1}^{K}\pi_{k}=1$, $0<\pi_{k}<1$, and $\theta_{k}=\left\{\pi_{k},\;\mu_{k},\;\Sigma_{k}\right\}$ denotes the parameter set in the *k*th Gaussian component, $\Xi =\left\{\theta_{1},\cdots ,\theta_{K}\right\}=\left\{\pi_{1},\mu_{1},\Sigma_{1},\cdots ,\pi_{K},\mu_{K},\Sigma_{K}\right\}$ denotes the vector of the GMM parameters. The mean vector $\mu_{k}$ and the covariance matrix $\Sigma_{k}$ specify an unknown multivariate Gaussian distribution $p(x_{i}|\theta_{k})$, whose probability density function can be formulated by:

(9) $$p(x_{i}|\theta_{k})=\frac{1}{\sqrt{{\left(2\pi \right)}^{m}\left|\Sigma_{k}\right|}}\exp\left[-\frac{1}{2}{\left(x_{i}-\mu_{k}\right)}^{T}\Sigma_{k}^{-1}\left(x_{i}-\mu_{k}\right)\right].$$

Expectation maximization (EM) algorithm, which consists of an E step and M step, is practically and extensively applied to estimate GMM parameters. The estimation process is the maximization process of the log-likelihood function defined as:

(10) $$\Xi =\underset{\Xi }{\arg\;\max}\left\{\log L(X|\Xi )\right\},$$

(11) $$L(X|\Xi )=\prod_{i=1}^{n}\sum_{k=1}^{K}\pi_{k}p(x_{i}|\theta_{k}),$$

where L(X|Ξ) is the likelihood function of X. Given an initial parameter set $\Xi^{\left(1\right)}$, EM algorithm in this way can produce a sequence of GMM parameters $\left\{\Xi^{\left(1\right)},\Xi^{\left(2\right)},\cdots ,\Xi^{\left(s\right)},\cdots \right\}$ by performing E step and M step successively, where *s* denotes the iteration times. The E step and M step iterate until they converge, which can be successfully carried out, as follows [21]:

E step: Calculate the posterior probability of *i*th training data point with *k*th component $C_{k}$ in the *s*th iteration:

(12) $$p^{\left(s\right)}(C_{k}^{i}|x_{i},\Xi^{\left(s\right)})=\frac{\pi_{k}^{\left(s\right)}p(x_{i}|\theta_{k}^{\left(s\right)})}{\sum_{k=1}^{K}\pi_{k}^{\left(s\right)}p(x_{i}|\theta_{k}^{\left(s\right)})}.$$

M step: Update $\theta_{k}=\left\{\pi_{k},\;\mu_{k},\;\Sigma_{k}\right\}$ of the *k*th component in the (*s*+1)th iteration by the following equations:

(13) $$\pi_{k}^{\left(s+1\right)}=\frac{\sum_{i=1}^{n}p^{\left(s\right)}\left(C_{k}^{i}|x_{i},\Xi^{\left(s\right)}\right)}{n},$$

(14) $$\mu_{k}^{\left(s+1\right)}=\frac{\sum_{i=1}^{n}p^{\left(s\right)}\left(C_{k}^{i}|x_{i},\Xi^{\left(s\right)}\right)x_{i}}{\sum_{i=1}^{n}p^{\left(s\right)}\left(C_{k}^{i}|x_{i},\Xi^{\left(s\right)}\right)},$$

(15) $$\sum_{k}^{\left(s+1\right)}=\frac{\sum_{i=1}^{n}p^{\left(s\right)}\left(C_{k}^{i}|x_{i}\right)\left(x_{i}-\mu_{k}^{\left(s+1\right)}\right){\left(x_{i}-\mu_{k}^{\left(s+1\right)}\right)}^{T}}{\sum_{i=1}^{n}p^{\left(s\right)}\left(C_{k}^{i}|x_{i},\Xi^{\left(s\right)}\right)}.$$

## 3. Hierarchical Sampling Strategy Based Active Learning Framework

**Figure 1:**
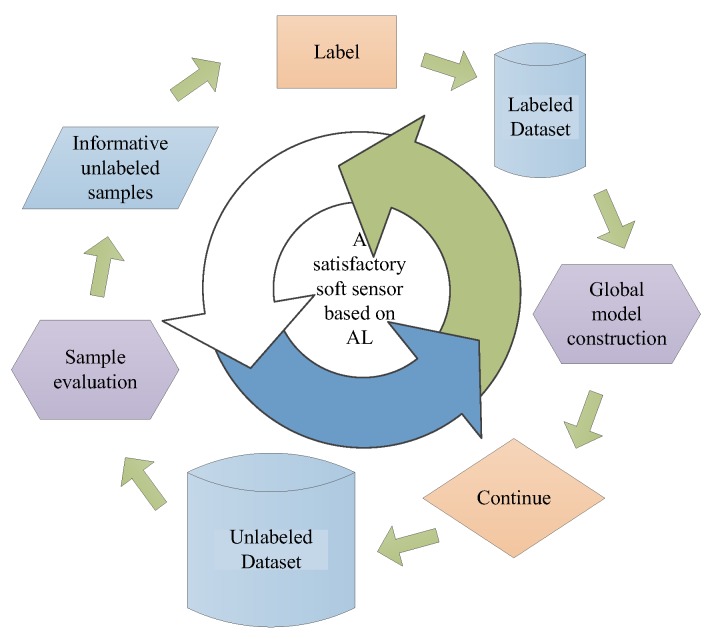
Flow diagram of active learning (AL) based soft sensor modelling process.

AL strategy has been developed and introduced to traditional sampling procedure in order to reduce the sampling bias resulted from random selection for unlabeled samples, which is shown in Figure 1. However, traditional AL based soft sensing cannot be able to make fully use of the spatial information between process samples, thus, in this section, the HC method and adaptive sampling strategy are introduced into the AL framework.

### 3.1. Hierarchical Clustering Algorithm

The HC method has been proven to be valuable to the data clustering. A tree of HC can be obtained by calculating the similarity of different clusters. In the clustering tree, the data samples with different characteristics are the low level of the tree, and the top level of the binary tree can be seen as the root node of the cluster. Additionally, the farther the distance on the cluster tree is, the less similar the two samples are. It has been studied to fully consider the special information between samples during the clustering process. 

The first important step of the HC algorithm is to calculate the distances between the data samples. As the most common distance measurement method, the Euclidean distance has been widely introduced to calculate the absolute distance between all given data points in the multidimensional space, which is defined by:

(16) $$d\left(x_{1},x_{2}\right)=\sqrt{\sum_{i=1}^{m}{\left(x_{1i}-x_{2i}\right)}^{2}},$$

where $x_{1}=[x_{11},x_{12},\cdots ,x_{1m}]$ and $x_{2}=[x_{21},x_{22},\cdots ,x_{2m}]$ represent the data points with *m*-dimension.

Another crucial task is the combination of different clusters. In this study, ward-linkage method, which aims to minimize the total variance of the clusters being merged, is employed to cluster combination [31,32]. The pair of data clusters that lead to the minimum increase in total produced variance, or the error sum of squares (ESS), are selected to merge at each union step to implement this method.

When considering the dataset ${\left\{x_{i}\right\}}_{i=1}^{n}$ in one-dimensional space, the variance is expressed, as follows:

(17) $$Var\left(x\right)=\frac{1}{n}\sum_{i=1}^{n}x_{i}^{2}-\frac{1}{n^{2}}{\left(\sum_{i=1}^{n}x_{i}\right)}^{2},$$

where n is the number of points. Subsequently, ESS is usually given by the following functional relation [31]:

(18) $$ESS=\sum_{i=1}^{n}x_{i}^{2}-\frac{1}{n}{\left(\sum_{i=1}^{n}x_{i}\right)}^{2}.$$

In ward-linkage cluster, the ESS value of the newly obtained cluster being merged is taken as the similarity of two clusters, which can be formulated as:

(19) $$ESS\left(c_{1},c_{2}\right)={\sum_{x_{i}\in c_{1}\cup c_{2}}d\left(x_{i},{ο}_{c_{1}\cup c_{2}}\right)}^{2},$$

where $x_{i}$ represents any data sample of two clusters before merging, $c_{1}$ and $c_{2}$ is a pair of clusters, ${ο}_{c_{1}\cup c_{2}}$ is the central data point of the new cluster, and $d(x_{i},o_{c_{1}\cup c_{2}})$ is the Euclidean distance between each sample $x_{i}$ to ${ο}_{c_{1}\cup c_{2}}$. In this way, the clusters with high similarity in measured characteristics are merged, and the complete hierarchical structure can be obtained by repeating the union process.

**Figure 2:**
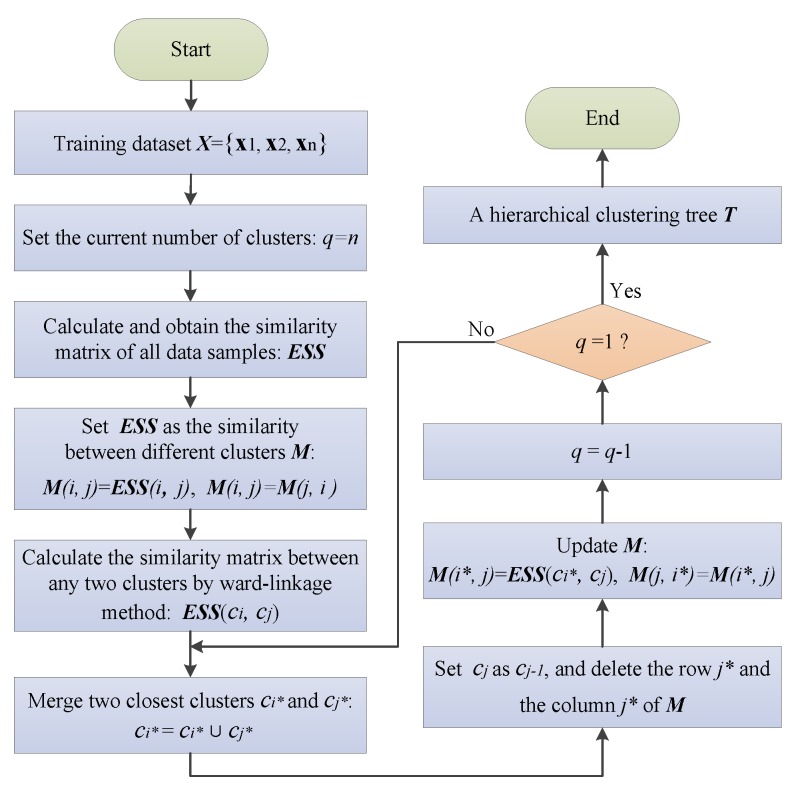
Flow diagram of the hierarchical clustering (HC) algorithm.

Figure 2 shows the implementation steps of the HC algorithm in detail.

### 3.2. Adaptive Sampling Strategy

A binary tree can represent the HC results, and the adaptive sampling based AL strategy is introduced into the smart soft sensor modelling. It aims to adaptively remove some redundant subtrees that were composed of those nodes that are homogeneous in HC tree according to a certain criterion. Here, the combined process is denoted as pruning. It aims to find an optimal pruning with minimum classification error and selected the most uncertain and informative samples for model training through an iterative process. Those samples that have less similarity with the labeled dataset are generally preferred as they have much useful information. Data sampling probability can effectively be reduced in regions of the space that already have labeled samples with relatively large numbers, which fully considers and makes use of the sample spatial information as compared to random selection (RS) and other sampling strategies.

Given the labeled dataset as $\{X_{L}\}\in R^{n_{l}\times m}$ and unlabeled dataset as $\{X_{U}\}\in R^{n_{u}\times m}$, where m denotes the number of measured process variables, $n_{l}$ and $n_{u}$ denote the numbers of the labeled data points and unlabeled data points, respectively, usually $n_{l}\ll n_{u}$ holds. Suppose the HC tree T has $n_{u}$ leaves, the number of data points in a node v∈T is expressed as $n_{v}$. A weight of the node is the proportion of the sample points in $T_{v}$, which can be represented as:

(20) $$p_{v,c}=\frac{n_{v,c}}{n_{v}},$$

where c=1,2,⋯,k is all possible classes and $n_{v,c}$ is the number of points that belong to class c. Generally, the class c with the maximal probability can be taken as the classification results of corresponding nodes. However, $n_{v,c}$ is sometimes small, and it might result in serious classification errors since the obtained classification probability $p_{v,c}$ has no enough robustness.

The generalization bounds are used to assess the quality of probability estimates in order to address this issue. When considering any given time t, we introduce a confidence interval $\left[p_{v,c}^{LB},p_{v,c}^{UB}\right]$ to replace $p_{v,c}$ by associating with each node v and class c [32]:

(21) $$p_{v,c}^{LB}=\max\left(p_{v,c}\left(t\right)-\Delta_{v,c}\left(t\right),0\right),$$

(22) $$p_{v,c}^{UB}=\min\left(p_{v,c}\left(t\right)+\Delta_{v,c}\left(t\right),1\right),$$

where $\Delta_{v,c}\left(t\right)=\frac{d_{v}\left(t\right)}{n_{v}\left(t\right)}+\sqrt{\frac{d_{v}\left(t\right)p_{v,c}\left(t\right)\left(1-p_{v,c}\left(t\right)\right)}{n_{v}\left(t\right)}}$, $d_{v}\left(t\right)=1-\frac{n_{v,c}\left(t\right)}{n_{v}\left(t\right)}$.

If it incurs at most β times as much as any other classes when class c is taken as the class of the node v:

(23) $$\left(1-p_{v,c}^{LB}\left(t\right)\right)<\beta \cdot \left(1-p_{v,c^{\prime }}^{UB}\left(t\right)\right),\;\forall c^{\prime }\neq c.$$

We consider class c to be an admissible class for node v, which implies that (v,c) is admissible at time t. In this study, we set β=2, in which case:

(24) $$p_{v,c}^{LB}\left(t\right)>2p_{v,c^{\prime }}^{UB}\left(t\right)-1,\;\forall c^{\prime }\neq c.$$

For any node v, several different classes may meet this criterion at time t. It is necessary to determine which class is chosen to be the optimal class for node v. In order to select the optimal class of nodes, the admissibility of all classes of nodes are first calculated, and then choose the class with the greatest probability as the optimal class to which the node belongs among all admissible classes.

The adaptive pruning strategy aims to combine similar subtrees and find an effective pruning in order to minimize the classification error as much as possible, which is directly related to the output of classification results. The classification error can be defined as:

(25) $${\tilde{\varepsilon }}_{v,c}\left(t\right)=\left\{ \begin{array}{ll} 1-p_{v,c}\left(t\right) & ,\;if\;\left(v,c\right)\;is\;admissible\\ 1 & ,otherwise \end{array}\right..$$

Generally, it starts from the root node and toward the leaves, evaluating whether the child nodes should replace its parent node if all of the descendants of the node are able to replace their parents. For each node of HC structure, its class and error are calculated. If it satisfies:

(26) $${\tilde{\varepsilon }}_{v,c}>{\tilde{\varepsilon }}_{v_{p},c_{p}}+{\tilde{\varepsilon }}_{v_{q},c_{q}},$$

where nodes $v_{p}$ and $v_{q}$ are the child nodes of v, in such cases, we can replace node v with its child nodes $v_{p}$ and $v_{q}$, which aims to reduce the overall classification error.

Once the optimal pruning is accomplished, a classification result can be obtained with minimum error. Subsequently, it can query some informative samples to refine $p_{v,c}$ in the iteration procedure, which further reduces the classification error. In this study, the AL strategy is introduced to effectively select queried samples for labeling. Normally, the node v with the minimal value of $p_{v,c}^{LB}$ is chosen to select samples for querying, and then one child node of v is chosen according to its node division. Repeat these two steps until the informative sample is selected and labeled.

After an iterative sampling process, the most dissimilar samples can be selected. As a result, the cost of human efforts and time for labeling can be greatly reduced. With the labeled dataset enlarged, $p_{v,l}$ the value increases while the confidence of classification is improved.

**Figure 3:**
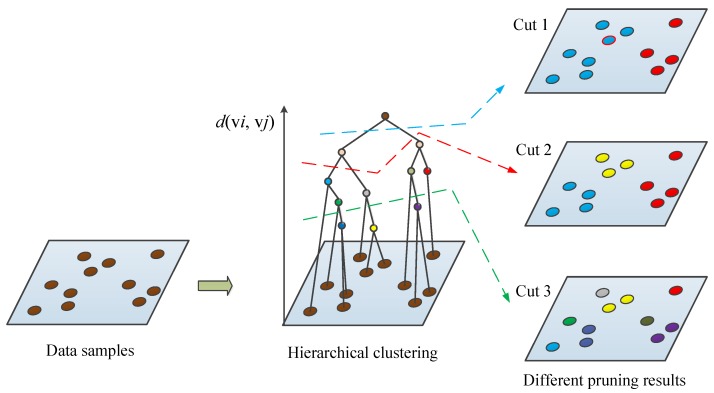
Flow diagram of HC process and different pruning results.

Figure 3 presents a schematic illustration of the HC tree and different pruning strategy. Algorithm 1 summarizes the proposed hierarchical sampling strategy under the AL framework.

**Algorithm 1.** The proposed hierarchical sampling strategy under AL framework.**Input**: a HC tree of n unlabeled data samples; iteration step $n_{s}$ **Process:**
1:Repeat following steps until labeled samples are enough for high-quality soft sensing or all unlabeled samples are labeled.2:Choose the node v∈T with minimal value of probability $p_{v,c}^{LB}$, and replace node v with its child nodes $v_{p}$ and $v_{q}$ if it satisfies ${\tilde{\varepsilon }}_{v,\;c}>{\tilde{\varepsilon }}_{v_{p},c_{p}}+{\tilde{\varepsilon }}_{v_{q},c_{q}}$.3:Choose one of the child nodes z in the same way, until there are no child nodes, then an informative sample x is selected.4:Update $p_{u,\;c}^{LB}$ of all nodes u∈T.5:Repeat step2 to step4 until $n_{s}$ unlabeled samples are selected.6:Query the labels of $n_{s}$ selected data points, and then configure the selected dataset $x^{s}$.7:Update the labeled dataset as $x_{new}^{l}\leftarrow [x^{l}+x^{s}]$, $y_{new}^{l}\leftarrow [y^{l}+y^{s}]$, the unlabeled dataset $x_{new}^{u}\leftarrow [x^{u}-x^{s}]$.
**Output**: Newly labeled dataset $x_{new}^{l}\leftarrow [x^{l}+x^{s}]$, $y_{new}^{l}\leftarrow [y^{l}+y^{s}]$.

## 4. Ensemble GPR Modelling Method Under AL Framework

Traditional soft sensing that is based on the AL strategy only constructs a global model for quality prediction, as shown in Figure 1, it usually ignores the multiphase and multistage characteristics of complex chemical processes. Therefore, a novel smart soft sensing technique with an AL strategy based on ensemble learning can be developed for better prediction performance. To guarantee the prediction capability of each ensemble sub-model, the GMM method is applied to obtain a set of local domains from updated training samples. Subsequently, sub-models can be built from different datasets, which, if applied effectively, could highly enlighten the generalization performance of soft sensing model. Besides, BIC criterion is chosen to determine the optimal number of Gaussian components, as it tends to establish a great structure for GMM model [22], which can be formulated, as:

(27) BIC=−2logL(X|Ξ)+3Klog(N),

where N denotes training data number, K denotes component number, and L(X|Ξ) represents the maximal values of the log-likelihood function. BIC aims to balance generalization performance with GMM model complexity and the model with the lowest BIC value is preferable.

In this paper, the GPR modelling method is chosen for ensemble model construction due to its better generalization behavior since the proposed AL method has no restrictions on the selected data model structure. In such cases, several GPR sub-models are driven by local datasets. Further, the posterior probabilities for each arbitrary observation $x_{q}$ with respect to all different phases can be formulated, as follows, since we apply Bayesian inference knowledge:

(28) $$p(C_{k}|x_{q},\Xi )=\frac{\pi_{k}p(x_{q}|\theta_{k})}{\sum_{k=1}^{K}\pi_{k}p(x_{q}|\theta_{k})}.$$

Afterwards, the localized GPR models are adaptively incorporated to an ensemble inferential model with posterior probability by applying the finite mixture mechanism. Usually, the final online estimation of key variable is the weighted combination of each individual, which is formulated, as follows:

(29) $$y_{p}=\sum_{k=1}^{K}y_{q}^{k}p\left(C_{k}|x_{q},\Xi \right),$$

where $x_{q}$ represents the new observation of test samples, $C_{k}=\{x^{k},y^{k}\}$, k=1,2,⋯,K represents *k*th process phases, and $y_{q}^{k}$ represents the local output.

**Figure 4:**
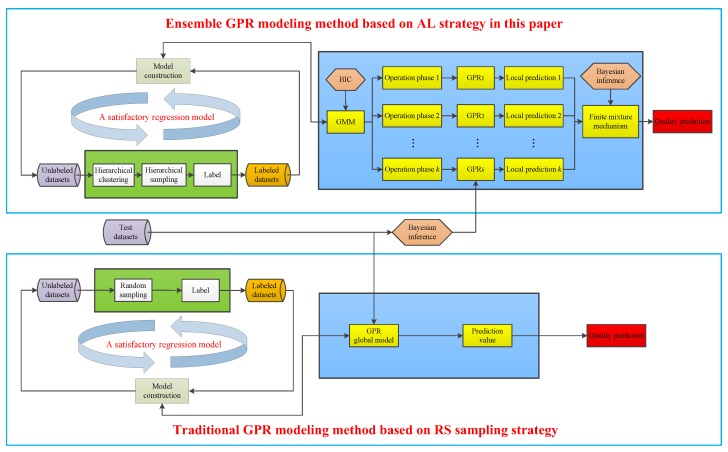
Comparisons of soft sensor modelling method between random selection (RS) sampling strategy and AL strategy proposed in this paper.

Figure 4 illustrates the comparisons between traditional global GPR modelling method based on RS sampling strategy and ensemble GPR modelling method based on the AL strategy presented in this paper.

## 5. Case Study

### 5.1. Process Introduction

**Figure 5:**
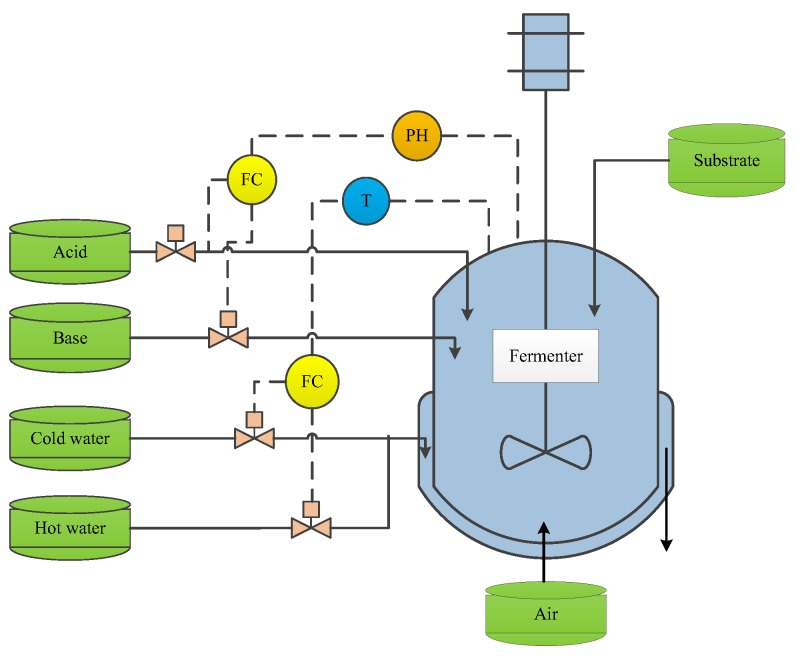
Schematic diagram of penicillin fermentation process (PFP).

The purpose of this section is obviously to prove the feasibility and superiority of the smart soft sensing method. Penicillin fermentation process (PFP) is traditionally regarded as a typical chemical process with nonlinearity, time-varying, dynamic, multi-batch, and other characteristics, which has been widely applied as a benchmark process in order to evaluate the effectiveness of soft sensor modelling methods. There are three physiological stages: cell growth, penicillin synthesis, and cell autolysis stage. For illustration, Figure 5 shows the detailed flowchart of the PFP. During the cultivation process, many factors, such as temperature, PH, sterile substrate, acid/base and cold/hot water flow rates, and dissolved oxygen concentration, can make a difference to penicillin production [13,33,34]. It is significantly important for humans to monitor and predict the penicillin concentration. However, there are many difficulties on penicillin measurement in a direct way due to the cost of hardware sensors. Soft sensor development is an effective solution for realizing the real-time estimation of penicillin concentration.

A simulator, named PenSim, has been proposed and widely applied in order to simulate the PFP under different operating conditions [34]. We can easily and effectively collect process data samples of PFP via PenSim platform. The Process Modeling, Monitoring, and Control Research Group of Illinois Institute of Technology developed PenSim, which is available at the website: http://simulator.iit.edu/web/pensim/index.html. A total of 16 process variables can be measured in the simulation plant. Generally, multidimensional datasets with more input variables contain abundant process information that makes a difference to informative model construction. However, some undesired problems, such as information redundancy and complex model structure, may also arise concerning the informative model based soft sensors. Less input variables give litter process information and that based models may lead to inaccurate predictions. In this case, we select seven input variables according to the experience of process engineers, which are enlisted in Table 1. For soft sensor development, 800 data samples are extracted under the normal operation condition, and then partitioned into two parts: 400 training samples for model establishment and remaining 400 test samples for model evaluation. For simulating the case of a little number of labeled samples, only 2% labeled samples (eight samples) are assumed. Subsequently, the other 392 data samples are unlabeled, and each sample only has the values of seven input variables without quality variable value.

### 5.2. Performance Evaluation of the Proposed AL Strategy

For the AL strategy, in this study, we set the learning step as 20 points, which means that 20 unlabeled samples are assigned with their real labels and become a part of labeled training dataset in each learning iteration. Under AL framework, all of the unlabeled candidates will be queried and labeled after a total of 20 iterations. However, we can stop the iteration process in advance when the soft sensors have satisfactory estimation accuracy, as it is unnecessary to update training dataset. Furthermore, two different global GPR model based soft sensors are developed for performance comparisons, which are designed with the RS strategy and hierarchical sampling based AL strategy, respectively. Here, the following root-mean-square error (RMSE) is traditionally used for an assessment of the soft sensing fit:

(30) $$\mathrm{RMSE}=\sqrt{\frac{1}{n}\sum_{i=1}^{n}{\left(y_{i}-{\hat{y}}_{i}\right)}^{2}},$$

where n is the number of test data, and $y_{i}$ and ${\hat{y}}_{i}$ denote the real and estimated values of *i*th test data point, respectively. The prediction accuracy and reliability of soft models to be tested can be greatly reflected by RMSE. Besides, 10 simulation times are carried out for two sample selection strategies, while the RMSE value is the mean value of 10 experiments in each iteration.

**Figure 6:**
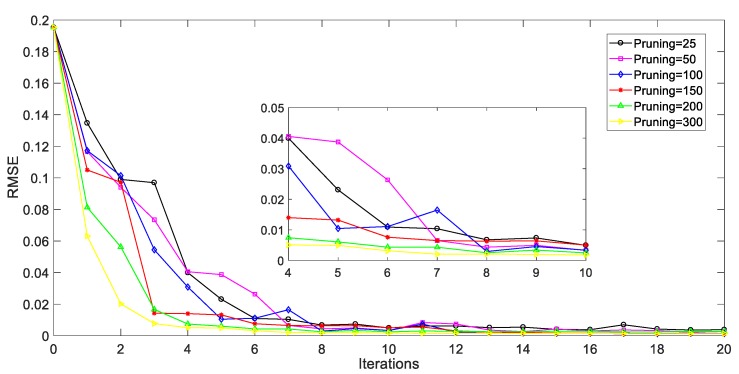
Prediction performance of soft sensors with AL strategy under different pruning results during iteration.

In our study, the HC method is introduced to explore the spatial information of all unlabeled samples that were collected in PFP. On the clustering tree, samples and their neighborhoods that sequentially merged in the same spatial level share the similar process information. The higher two samples are connected on the tree, the more dissimilar they are. The hierarchical sampling based AL strategy is then proposed in order to evaluate each unlabeled sample and selected the most valuable ones for labeling. The pruning results are relevant to the estimation ability of the AL based soft sensors. Figure 6 shows the prediction performance of the soft sensors under different pruning results. Here, RMSE values of penicillin concentration are used for model performance evaluation. Generally, an informative and detailed pruning of the HC tree makes a significant difference to superior generalization ability, as seen in Figure 6. However, it might also lead to the high costs of clustering and pruning as well as complex model structure. The number of pruning is recommended to be set as 150 when considering the balance of estimation performance and model complexity, in this case. 

**Figure 7:**
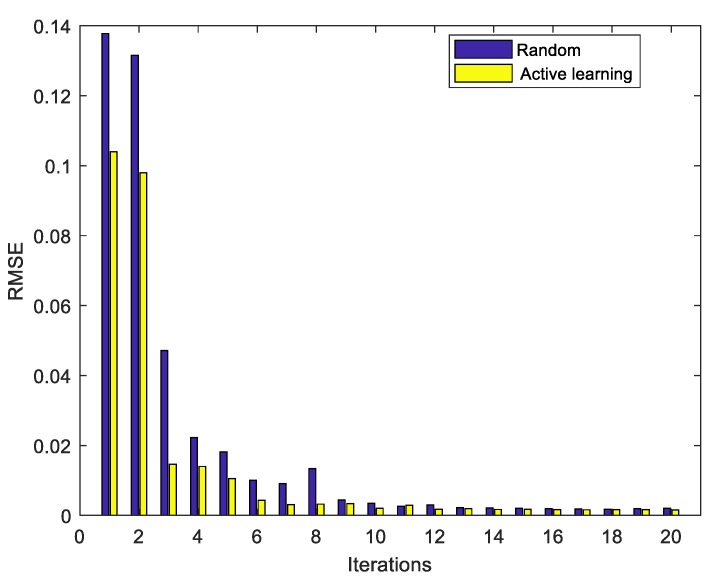
Root-mean-square error (RMSE) values of penicillin concentration under AL and RS sampling strategies during iteration.

Figure 7 demonstrates the RMSE values for global GPR models that are developed with AL and RS strategy in each iteration, respectively. The results reveal that, for both sampling strategies, GPR predictors are tested for their estimation accuracy and performance by selecting some unlabeled samples. This is because modelling space is significantly enlarged by labeling unlabeled samples and adding them into labeled dataset pool in each iteration. Obviously, those GPR models with AL strategy perform much better than those that were developed with the RS strategy, as the RMSE index values of the former are much smaller than that of the later in all iterations. The AL based GPR modelling method selects the most dissimilar samples that have the most valuable information for processes, while it cannot be guaranteed under the RS strategy. The estimation performance of GPR models that are based on RS may not be improved; if handled badly, it would even be deteriorated. Because there exists a potential risky issue that some samples in bad quality, usually with environment noise, measurement error, or variable mismatch, may be sampled and labeled for modelling, which might distort the structure of the soft sensing models. 

It can be easily observed that the RMSE index values of AL based GPR soft models heavily decrease after two iterations, which is the same as the RS based models. It means that, for both sampling strategies, the estimate performance has been converged after the first two iteration steps because of the enlarged modelling space. However, the AL strategy converges faster than the RS strategy, especially in the third iteration step. This result shows that less unlabeled samples are recorded for selection and labeling under AL framework. Moreover, during the first three iterations the converge speed that we can find in Figure 6 is greatly higher than that one during the later iterations. Thus, we can infer from the result that the estimation ability of AL based soft model is effectively enlightened during the first three iterations, but the improvement trend after that is greatly limited during the third to twentieth iterations. As iteration number increases, the additional information of the selected samples in later iterations could hardly have a significant impact on high-quality soft sensor development.

**Figure 8:**
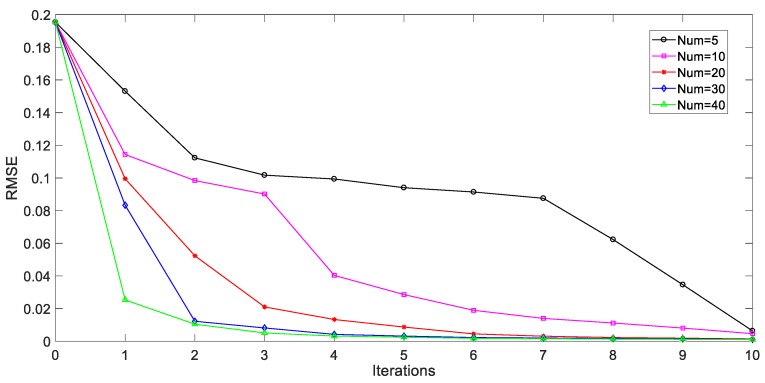
RMSE values of penicillin concentration for the AL based soft sensors under different number of selected samples.

Besides, the influence of different query sample numbers upon AL based soft sensors is also examined in this study. For this purpose, various AL steps for soft sensing are selected in each iteration, which are set as 5 to 40. Figure 8 illustrates a comparative testing result of the average index values under different point numbers to be labeled in each iteration. Smart soft models with a different number of selected data points are developed and compared under the same iteration. For example, after 10 iterations, the smart soft predictor with 40 learning steps, have queried and add all of the unlabeled samples into previous labeled dataset, while only 50 unlabeled samples are labeled for the soft predictor with five learning steps. In most cases, the prediction ability of the smart sensor is enhanced when more unlabeled data points with useful information are selected and queried in each iteration. However, it also increases the computational burden and needs more human efforts for sample annotation process to model construction.

**Figure 9:**
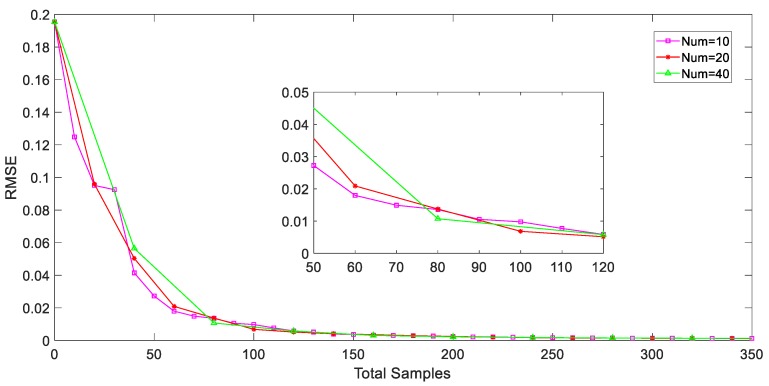
RMSE values of penicillin concentration for the AL based soft sensors under different total numbers of labeled samples.

Furthermore, we intend to research the estimation performance of some different soft sensors that are based on AL with the same total number of labeled data points. With the increase of labeled numbers, as shown in Figure 9, the converge speed of the soft sensors becomes higher in early iterations, while the additional process information of the remaining selected samples in later iterations could hardly make a big impact on generalization enhancement of the GPR model. Under AL framework, a desired GPR model can be constructed with less labeled data samples, which is significantly helpful in human effort reduction.

### 5.3. Prediction Results and Discussions

Two other different kinds of smart predictors are built with the updated training data in order to research the influence of the ensemble learning based on GPR model with a great number of unlabeled data samples. There are four soft sensors built in the present work:

(1) GPR (GPR based on RS strategy): To iteratively select unlabeled samples for labeling with RS sampling strategy, and to construct a global GPR model.
(2) EGPR (ensemble GPR based on RS strategy): Firstly, to iteratively select unlabeled samples for labeling with RS sampling strategy, to further construct local GPR models on different regions divided by GMM method, and finally to integrate the base predictors by applying the Bayesian fusion strategy.
(3) AL-GPR (GPR based on AL strategy): To iteratively select unlabeled samples for labeling with hierarchical sampling based AL strategy and construct a global GPR model.
(4) AL-EGPR (ensemble GPR based on AL strategy): the proposed method.

Table 2 describes and compares the characteristics of all different soft sensors.

In addition to RMSE, the tracking precision (TP) criterion is also applied to assess the generation capabilities of these soft sensors, which is obtained by:

(31) $$\mathrm{TP}=1-\frac{\sigma_{error}^{2}}{\sigma_{true}^{2}},$$

where $\sigma_{true}^{2}$ denotes the variance of the true value and $\sigma_{error}^{2}$ denotes the variance of the error between the output value and true value. TP is the variance correlation between the estimation error and the actual outputs, which can be applied to measure the tracking performance of the regression model. The soft sensing model with the higher TP value is preferable.

**Figure 10:**
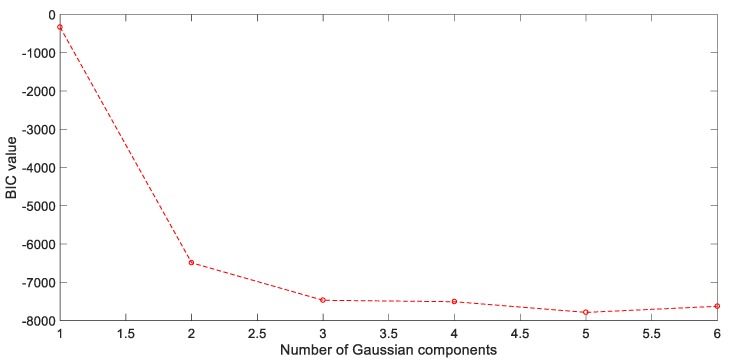
Bayesian information criterion (BIC) performance against variation of Gaussian components.

In this case, GMM is used to divide the updated process dataset into three subsets. Subsequently, the BIC criterion is applied for structure optimization to avoid the model over-fitting and contribute to data interpretation ability enhancement. The BIC value decreases gradually while Gaussian component number increases, as shown in Figure 10. However, further increases of K value do not cause further decreases of the BIC value. Combined with the prior knowledge of PFP, which consists of three physiological stages, one can be judged that the most optimal component assignment should set as 3.

The estimation results of predictors for penicillin concentration after the 3rd and the 7th iterations are tabulated and compared in Table 3 for detailed analysis on the performance of different soft models. From the results of the RMSE and TP values, the AL-EGPR model based soft sensor obtains the best generalization performance, as it has the lowest RMSE value and the highest TP value for penicillin concentration prediction. When comparing the RMSE and TP values between AL based and RS based GPR models, it can be easily found that the modelling ability becomes highly improved because the informative unlabeled samples are considered. By introducing the AL based hierarchical sampling strategy, the global GPR model and ensemble GPR model can all achieve higher prediction accuracy than other two GPR models with RS strategy after the 3rd and the 7th iterations. When comparing with the AL-GPR model, the AL-EGPR model under the ensemble learning framework performs much better and obtains the smaller error, since it partitions the updated dataset into several subsets for sub-predictor construction. In general, for two different data sampling strategies, the estimation accuracy and model capability can both be improved when iteration step increases. Meanwhile, the models after the 7th sampling iteration can achieve a better prediction performance than those that were developed after the 3rd sampling iteration. It should be noticed that, similar to the previous case, here, also, local GPR based soft sensors yield lower RMSE values and higher TP values, which performs better than the single GPR predictors under ensemble learning framework. However, in the present case, there is a relatively small reduction in RMSE, which can be attributed to the enough samples for labelling and model training. The recommended soft sensor shows its superiority and high performance in modelling the uncertainty of estimation under the complex measurement environment. 

**Figure 11:**
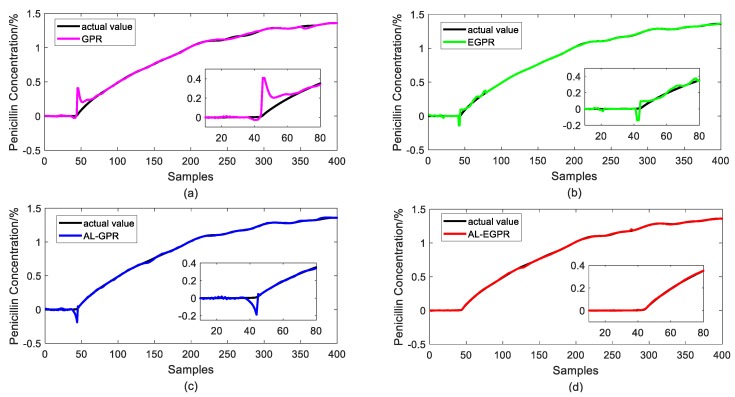
Prediction results of test samples for four different soft sensors after the 3rd iteration. (**a**) Gaussian process regression with random selection strategy (GPR) model; (**b**) ensemble Gaussian process regression with random selection strategy (EGPR) model; (**c**) Gaussian process regression based on active learning (AL-GPR) model; and, (**d**) ensemble Gaussian process regression based on active learning (AL-EGPR) model.

**Figure 12:**
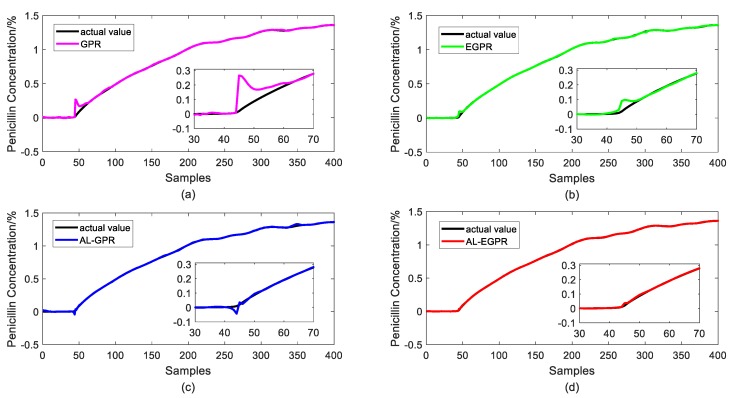
Prediction results of test samples for four different soft sensors after the 7th iteration. (**a**) GPR model; (**b**) EGPR model; (**c**) AL-GPR model; and, (**d**) AL-EGPR model.

Figure 11 and Figure 12 illustrate the prediction results of test samples by these four soft sensors after the 3rd iteration and the 7th iteration, respectively, to show the prediction performance more intuitively. In Figure 11, the GPR predictor presents the worst prediction performance on account of its RS strategy and global model structure. By contrast, EGPR and AL-EGPR, on the basic of ensemble model structure, further enhance the generalization capability by partitioning the training data into isolated regions for local modelling. The two ensemble model based soft sensing strategies are both able to track the main trend of the penicillin concentration. In comparison, the proposed AL-EGPR soft sensing model as well as AL-GPR model performs much better than GPR and EGPR do, as its prediction output results are closer to the real values. The similar conclusion can be drawn from the prediction results in Figure 12. Generally, the modelling space is enlarged, and it contributes to developing a satisfactory soft sensor with high prediction accuracy. In addition, the dataset partition based ensemble learning is particularly effective to handle the multiphase processes with high complexity, and thus further enhances estimation behavior of regression model. 

**Figure 13:**
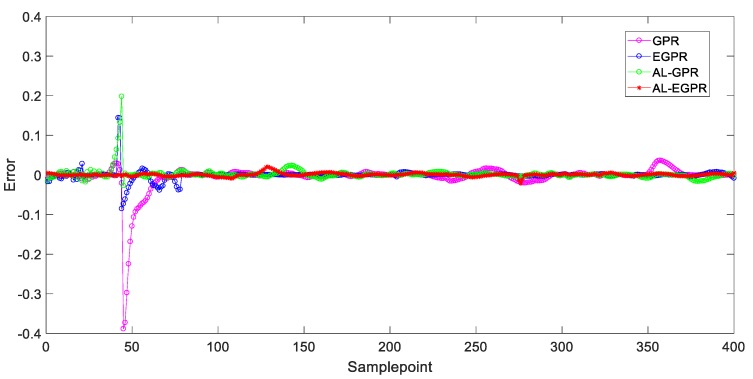
Prediction error of test samples for four different soft sensors after the 3rd iteration.

**Figure 14:**
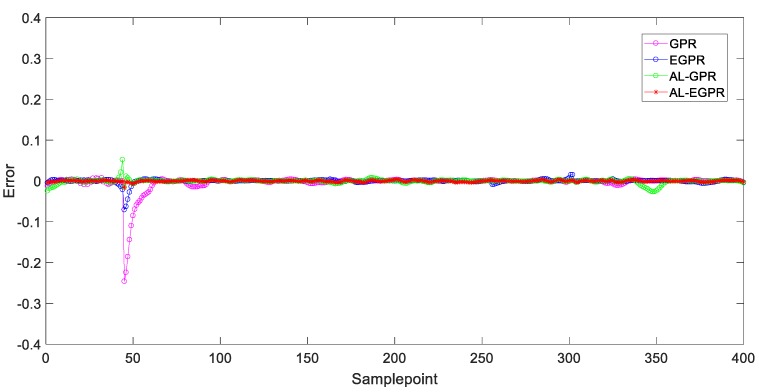
Prediction error of test samples for four different soft sensors after the 7th iteration.

The estimation error results of four different manners after the 3rd and the 7th iterations are given in Figure 13 and Figure 14, respectively, to reveal the effectiveness of the proposed soft sensor further. The closer the error curve is to the zero line, the more accurate the prediction is. By comparing these four prediction error results, we can readily conclude that the global GPR model that is based on RS strategy performs worst among the four soft sensing models and the proposed soft sensor modelling under ensemble learning framework further provides a more accurate prediction on the basis of AL-GPR with active learning strategy. 

## 6. Conclusions

The data produced from any element of the industrial process drive the implementation of Industry 4.0. Our basic idea is to process large amounts of data with smart data-driven soft sensors that can extract useful process information that is contained in labeled data as well as unlabeled data by means of machine learning and artificial intelligence. The hierarchical sampling based AL strategy has been proposed and introduced into the traditional ensemble GPR modelling method for soft sensing. Under the AL framework, the most representative and uncertainty samples with additional process information are selected and labeled to enlarge the labeled dataset and, thus, lots of human efforts and time costs that are related to labeling samples can be saved. We use the hierarchical sampling strategy rather than the RS to accelerate the convergence process and maximize the prediction capacity of ensemble models with the minimal labeled samples. We have evaluated the recommended soft sensor in penicillin fermentation process, showing that at least half of the time and human resource can be saved.

The exploitation of the hierarchical sampling based AL strategy can be a boost for unlabeled data analysis and processing. It is remarkably effective for engineers to handle the control and modelling problems with a limited number of labeled samples. Another outstanding advantage of our smart soft sensing technique is that the ensemble learning based GPR model can significantly address the strong nonlinear, highly varying, and multiphase characteristics of complex industrial processes. Hopefully, these contributions would provide the leaders of Industry 4.0 with a novel data analysis and modelling method for achieving a better performance of sensors under a small percentage of labeled process data.

## Figures and Tables

**Table 1:** Input variables selected in PFP.

Input Variables	Description	Unit
*u*_1_	Culture volume	L
*u*_2_	Agitator power	W
*u*_3_	PH	-
*u*_4_	Substrate feed temperature	K
*u*_5_	Fermenter temperature	K
*u*_6_	Substrate feed rate	g/h
*u*_7_	Aeration rate	L/h

**Table 2:** Characteristics of different soft sensor modelling methods.

Methods	Sample Selection	Learning
GPR	Random	Global
EGPR	Random	Ensemble learning
AL-GPR	Active learning	Global
AL-EGPR	Active learning	Ensemble learning

**Table 3:** Prediction performance indicators of different modelling methods after the 3^rd^ iteration and the 7^th^ iteration.

Method	After the 3rd Iteration		After the 7th Iteration	
	RMSE	TP	RMSE	TP
GPR	0.0472	0.9894	0.0106	0.9995
EGPR	0.0155	0.9988	0.0069	0.9997
AL-GPR	0.0143	0.9991	0.0065	0.9997
AL-EGPR	0.0039	0.9998	0.0017	0.9999

## Abbreviations

The following abbreviations are used in this manuscript:

AI	artificial intelligence
AL	active learning
AL-GPR	Gaussian process regression based on active learning
AL-EGPR	ensemble Gaussian process regression based on active learning
ANN	artificial neural network
BIC	Bayesian information criterion
EGPR	ensemble Gaussian process regression with random selection strategy
EM	expectation maximization
FCM	fuzzy C-means
GMM	Gaussian mixture model
GP	Gaussian process
GPR	Gaussian process regression
HC	hierarchical clustering
ML	machine learning
PCR	principal component regression
PFP	penicillin fermentation process
PLS	partial least squares
RMSE	root-mean-square error
RS	random selection
SVM	support vector machine
TP	tracking precision
